# Tourist perceptions, motivations and expectations when interacting with African lion (*Panthera leo*) cubs

**DOI:** 10.1017/awf.2024.63

**Published:** 2024-12-16

**Authors:** Ann Wilson, Clive JC Phillips

**Affiliations:** 1 Applied Behavioural Ecology and Ecosystem Research Unit (ABEERU), Department of Agriculture and Environmental Sciences, University of South Africa, Private Bag X6, Florida 1710, South Africa; 2 Institute of Veterinary Medicine and Animal Sciences, Estonian University of Life Sciences, Kreutzwaldi 1, 51006 Tartu, Estonia; 3 Curtin University Sustainable Policy (CUSP) Institute, Curtin University, Australia

**Keywords:** animal interactions, animal welfare, education, expectations, impact, lion cubs

## Abstract

Tourism wildlife interactions are controversial, the debate hinging largely on the compromised welfare of the animals used. Despite this, lion cub (*Panthera leo*) interactions are popular, and there is a need to understand what motivates interactors to participate in the activity, their perceptions and expectations. We surveyed the attitudes of 300 visitors to three lion cub interaction facilities in South Africa. Whilst 38% of interactors were aware of the controversy around lion cub interactions, 69% desired the experience regardless. It is widely assumed that lion cub interaction opportunities are big attractions, yet 74% of respondents said that they would still have visited if lion cub interactions were not offered. Whilst 84% of interactors felt that their expectations were met, 61% said that the interaction had no impact on them. Several of those interviewed interacted with multiple species, and 34% determined that their favourite engagement was with animals that interacted back voluntarily. Most of those interviewed chose the interaction for their children (69%). Whilst 58% felt the experience was educational, only 2% of these had learnt about the plight of lions in the wild. When asked to reflect on the welfare of the lion cubs they had interacted with, ‘Freedom from discomfort’ was seen as the most important factor, as well as ‘Freedom to express natural behaviour’. Interactions were viewed with a variety of emotions and generated a range of beliefs. We conclude that the findings can be used by facilities to better prepare visitors for the experience, ensuring that interaction animals are better able to serve in their role as ambassador representatives.

## Introduction

The use of wildlife in tourist interactions in zoos, wildlife parks and sanctuaries, remains controversial (Macdonald *et al*. 2017), with some suggesting that it allows for both locals and tourists to be informed about the plight of animals and their habitats (Higginbottom & Tribe 2004). Predator-focused tourism has been shown to have the capacity to support predator conservation, as long as there is both public and political support for management and monitoring bodies (Macdonald *et al*. 2017). Wildlife interactions vary from strictly regulated institutions to facilities that are poorly managed and unregulated. The latter may cause people to believe that animals used in tourism are exploited for human entertainment, with lion cub (*Panthera leo*) interactions being labelled as one of the “*cruellest activities*” tourists can participate in (World Animal Protection undated). A second controversy, specific to the use of lion cubs in South African tourism, centres around the post-interaction use of some lion cubs in the captive lion industry, including ‘canned’ lion hunting and the bone trade (Williams & ‘t Sas-Rolfes 2019).

In order for controversies over wildlife interactions to be quelled, it is clear that there should be benefits for the animals as much as there are benefits for the interactors. Ballantyne *et al.* (2011) summed up the positives for the wildlife tourist as being “*heightened awareness, appreciation of and reconnection with nature, personal rejuvenation and a realisation of personal responsibility for the state of the environment*”. Those for the wildlife were considered to include “*providing income for the ongoing protection and sustainable management of wildlife and wildlife habitats, encouraging visitors to make financial and non-financial contributions to environmental causes, providing socio-economic incentives for the conservation of natural resources and influencing tourists’ behaviour during their visit*”. These benefits are more for the species and their wild counterparts as opposed to the individual animal ambassadors themselves. Baird (2018) suggests that this is because best practice guidelines are generally designed for zoos and sanctuaries and do not take into account the varied husbandry and interaction conditions such ambassador individuals are exposed to.

Humans are naturally drawn to viewing and interacting with animals, both domesticated and non-domesticated (Fawcett & Gullone 2001). An emotional connection with animals is a humanistic characteristic within people’s connection to nature (Kellert 1983). This desire for close animal interactions suggests a romantic and anthropomorphic view of animals (Curtin 2005). Excluding wild animals (including in wildlife tourism interactions) from the lives of people can potentially upset their cognition, personality and inner life (Kellert 1983). However, when the media depict such interactions, they can easily influence the public’s perceptions of the animals, making them appear to be friendly, domesticated animals and less like the potentially dangerous animals they are (van der Meer *et al.* 2019). When expectations of interacting with wildlife are then not met, because of a misperception, it could have a profound effect upon the human experience (Van Manen 1990). The psychological benefits of participation in an activity are considered the primary reward, but increased understanding of the animals may also lead to a greater sense of environmental awareness (Schänzel & McIntosh 2000).

Individuals are attracted to a variety of animal species, a phenomenon which is applied in the tourism industry, where different species are selected by wildlife operators to attract certain types of visitors (Woods 2000). As outlined by Carr (2016a), an animal’s attractiveness to a person can occur as a result of their charismatic identity (Small 2011), large body size (Moss & Esson 2010), activeness (Puan & Zakaria 2007), rarity (Whitworth 2012) and/or strength (Sommer 2008). There is a complex interaction between these characteristics which makes them attractive to the visitor (Carr 2016b). Lions in western countries are regarded as the second most charismatic species in the world, after tigers (*Panthera tigris*), and the desired traits are beauty, impressiveness and dangerousness (Albert *et al.* 2018). Lion cubs are considered suitable for attracting visitors since they embody all of these traits as well as having the benefit of being juveniles, another attractive trait (Small 2012).

The mission of most zoos is to conserve species, provide educational opportunities, conduct research and exhibit animals for entertainment (Cain & Meritt Jr 1998). People also visit zoos for family or friend bonding time (Rajack & Waren 1996; Holzer *et al.* 1998), education (Andereck & Caldwell 1994), and entertainment (English Tourist Board 1983). Providing children access to a zoo exhibit allows them to experience animals in a novel and more educational and imaginative context than at home, thus it is common for adults to visit with young members of the family (Oxarart *et al.* 2013). Positive childhood experiences, such as observing, exploring and interacting with natural objects encourages conservation interests later in life (Vadala *et al.* 2007). Conversely, a zoo may be considered discordant because they attempt to educate and conserve wild animals at the same time as exhibiting them in captivity (Jamieson 1985). Many zoos have therefore been challenged to prove that they effect attitude or behaviour change, given these unnatural situations (Mason 2000). Children who visit zoos in a formal setting, such as with a school, appear to recognise the educational and conservation roles better, whereas those that visit with family tend to be more anthropocentric (Almeida *et al.* 2017).

Many adults accept the social remit of the modern zoo, which emphasises conservation and education functions, along with entertainment (Carr & Cohen 2011). The more animated an animal’s behaviour is at a zoo, the more human attention it attracts, potentially improving the learning experience (Altman 1998). Human-wildlife conflicts can be reduced with better public education (Carr & Broom 2018). It would seem therefore that interaction facilities have the potential to produce experiences to teach, not only the public, but also affected communities regarding the conservation plight of many endangered species.

Most zoo visitors consider themselves capable of assessing animal welfare, by observing enclosure style and animal behaviour (Melfi *et al.* 2004). However, aesthetic characteristics, which appeal to visitors, do not always imply benefits for the animals (Seidensticker & Doherty 1996), and most visitors do not observe the animals for a sufficient enough period of time to assess the meaning of observed behaviours (Melfi *et al.* 2004). Packer *et al.* (2018) found in a study involving gorillas (*Gorilla gorilla gorilla*) that visitors confidently expressed their judgements on how the gorillas were coping with the conditions within which they were living, through health and happiness indicators, whilst judgement on the way the gorillas were feeling was dependent upon the visitors’ emotional connection with the gorillas and their overall satisfaction with the visitation. Despite the confidence with which the visitors appear to assess welfare, the likelihood is that they are poor judges (Moorhouse *et al.* 2015). Human attitudes towards animals directly affect how well they provide for animal welfare (Serpell 2004) and, by association, their perceptions of welfare states. These human attitudes are affected by the attributes of the animal in question, the characteristics and experiences of the evaluator and an array of cultural factors.

Our aim was to better understand interactor perceptions, motivations and expectations when interacting with lion cubs. The first objective was to determine if current lion cub interaction controversies affected their decision to interact with the cubs; the second to ascertain the desirability of lion cub interactions compared to other animal interaction encounters; the third was to determine whether there was an association between pre-interaction expectations and post-interaction attitudes, and how children might influence these; the fourth sought to determine the educational outcomes associated with lion cub interaction encounters; and the fifth objective was to understand the interactors’ perception of the welfare of the lion cubs in question.

## Materials and methods

### Ethical approval

Ethical approval was granted for this study by the University of South Africa’s College of Agriculture and Environmental Sciences, Human Health Research Ethics Committee (reference number: 2016/CAES/106). Three legally operating South African lion cub tourism interaction facilities gave their approval for the study (names and location are anonymised). Each facility offered a slightly different interaction experience, additionally offering interactions with other animals and amenities, specific to each facility.

### Study facilities

Facility A was mostly frequented by South Africans due to its geographic location. Interactors were allowed to freely interact with a sister cohort of three cubs of between 34 and 100 days of age, depending on when the interaction was undertaken. The number of interactions per day was the lowest of the three facilities (mean: 24) and so interactors were able to experience the interaction at their ease and for as long as they so desired, usually lasting several minutes.

Facility B was well known to both the international and local market, including through tour operators. Interactions took place at set times and formed part of a tour, with individual interaction times generally not being limited but still keeping to a schedule. Interaction numbers per day (mean: 31) were slightly more than those experienced at Facility A. There was a mixed cohort of cubs but of very similar age, and petting was restricted mainly to the head and back. Other body positions were allowed if not aversive to the cub and acceptable to the handler. Cubs interacted with were aged between 34 and 193 days, depending on when the interaction took place.

Facility C was also known to the international and local market, making use of tour operators at times. Interaction times were limited due to the comparatively higher numbers of interactors (mean: 102), as was the extent of the interaction, only allowing the petting of the head and back. A mixed cohort of varied ages were available for interactions, with cubs aged between 57 and 271 days, depending on when the interaction was undertaken.

### Questionnaire design

There were 300 anonymous respondents for the questionnaire which was completed between March and September 2017. All adult (> 18 years) interactors who had completed their interaction experience were asked to participate during the attendance of the lead researcher (AW). Whilst only consenting adults were interviewed, they were questioned about their children’s experiences, which allowed these to be included in the results. Visitors were interviewed after the interaction had taken place to enable them to reflect upon the experience, something that would have been impossible prior to interacting. The first 100 respondents to consent and fully complete the questionnaire at each facility were included in the study as the questionnaire was deemed to have reached saturation at this point, with no new answers being recorded. Approximately 10% declined to be interviewed. Questions were asked by two interviewers, one experienced in the field (AW) and the other trained to do the work. Interviewers were used because it increased acceptance rate, the interviewers could seek clarification of responses, and it allowed respondents to seek clarification if necessary. Mean response time was approximately ten minutes. The interviewer remained neutral and impartial throughout the process by not influencing the respondent with ideas and suggestions nor participating in any debates around the questions. Apart from the demographic questions at the start of the questionnaire and four binary ‘yes/no’ questions, the remainder of the 18 questions were open-ended to allow for unexpected responses and accommodate the novelty of the topic. These were then classified through a process of coding (O’Cathain & Thomas 2004). Codes were generated from the themes of the responses received, and each identified code was clearly distinguishable from another. Gatekeeper permission was granted by the three facilities. Participants were allowed to withdraw from the interview at any point, however, no one did.

Part one of the questionnaire (Appendix 1; Supplementary material) requested demographic information: age (grouped into three broad age classes), continent where they lived (if they had lived on multiple continents, then the one they most associated themselves with), gender and current dwelling location (urban, suburban or rural environment). Part two dealt with controversies around lion cub interactions, and respondents were asked to provide their reasons for interacting if they were aware of such controversies (the controversies were not specifically discussed in a deliberate effort not to lead the respondents). Part three asked about their prior expectations and post-interaction impact. Part four attempted to determine what importance value the respondent placed on the lion cub interaction, including comparison with other animals interacted with. Part five examined whether children were involved in their experience and, if so, how did the interaction influence their experience. Part six looked at education during the interaction to determine what was learnt and Part seven asked how interactors perceived the welfare of the cubs they interacted with.

### Statistical analysis

Logistic regressions were applied to the binary response variables, such as yes/no responses. The glm() function in the stats() package R (R Core Team 2013) was used to generate a model with a logit-link function. Wald type 3 analysis of deviance testing was used to assess factor significance through the Anova () function using the car () package (Fox & Weisberg 2019). For multinomial responses, the multinom () function in the nnet () package (Ripley *et al.* 2016) was used to generate a model with a logit-link function. The open-ended questions were manually coded from qualitative responses. Again, Wald type 3 analysis of deviance testing was used to assess factor significance of the demographic factors, through the Anova () function using the car () package (Fox & Weisberg 2019). Only significant (*P* < 0.05) factors are reported in *Results*, together with tendencies (*P* < 0.10), whereas the remaining non-significant findings appear in Appendix 2 (see Supplementary material).

## Results

Most respondents were 31–50 years of age (61%), came from Africa (63%), and lived in suburbia (72%); 55% identified as female and 45% as male (Table 1).

**Table 1.** The demographic profile of the 300 respondents who participated in the questionnaire at the three lion cub interaction facilities, forming a part of the study to determine their perceptions, motivations and expectations with regards to interacting with lion cubs

Demographics		Respondents (n)	(%)
Age	18–30 years	77	25.7%
	31–50 years	183	61.0%
	> 51 years	40	13.3%
Continental association	Africa	188	62.7%
	Europe	50	16.7%
	North America	26	8.7%
	Asia	16	5.3%
	South America	12	4.0%
	Australasia	8	2.7%
Gender	Female	165	55.0%
	Male	135	45.0%
Dwelling	City	70	23.3%
	Suburbia	217	72.3%
	Rural	14	4.7%

Of the 300 respondents, 113 (38%) were aware of the controversy surrounding the practice of interacting with lion cubs. The coded reasons provided by 108 of the 113 participants who responded to the question on why they still interacted with the cubs despite being aware of such controversies included 74 respondents (69%) who indicated that they “*still wanted the experience, irrespective of the controversy*”. Fifteen (14%) said they had “*verified the facility for themselves prior to visiting*” and had felt the facility could not be associated with such controversy. Nine participants (8%) wished to “*give the facility the benefit of the doubt*” and so determined the situation for themselves. Eight participants (7%) felt secure in their decision to interact as a result of “*recommendations provided by others who had interacted previously*”, and two participants (2%) were “*dismissive of the controversy*” and questioned its validity.

Referring to the controversy around the welfare of cubs at such venues, respondent 84 stated that he “*didn’t want to go* [interacting] *because they have probably been petted excessively over the long weekend, but if not today, then when?*” Respondent 120 referred to the alternate controversy around the post-interaction life of a cub and rationalised his decision to interact by stating that “*we would pet lambs and we know where they end up*”. Respondent 46 did not allude to a specific controversy but stated that the “*experience is for my son despite the controversy*”.

Two hundred and twenty-one (74%) respondents indicated that they would have still visited the facility even had lion cub interactions not been offered, whilst the other 79 (26%) specifically visited to interact with the lion cubs. Four coded responses were derived from the 221 respondents as to why they had decided to visit the facility. Eighty-two respondents (37%) visited the facilities “*to experience the other wildlife*”. Sixty-two respondents (28%) specifically visited for the “*other facilities not associated with wildlife or lions*”, with the cubs becoming an addition to the day’s activities. Fifty-four (24%) were visiting for a general lion experience, not necessarily interacting with the cubs, and coded as “*other lion activities*”, and 22 (10%) were simply seeking something to experience for the day; “*an outing*”. These coded responses, as expected, were significant for the facility (*χ* ^2^_12_ = 95.1; *P* < 0.0001), given the very clear differences between them.

Two hundred and fifty-one (84%) of the 300 respondents felt that their expectations had been met through their interaction experience, whilst 49 (16%) did not. Five coded responses were derived from the 300 respondents in response to their expectations around interacting with the lion cubs. Most respondents, 196 (65%) felt that “*the interaction was the expectation*” while 36 (12%) “*expected to cuddle with smaller cubs but they were too big and/or rough*”. On the other hand, 31 (10%) respondents “*expected more action – cubs inactive and/or too small and/or time too short*”.

Respondent 28 “*expected the cubs to be younger so that they could be held on the* [her] *lap”*, while Respondent 33 reflected on a previous interaction that they had had, stating that it was a *“pity they were not small, as I hoped to pick them up, as* [they had] *in Taiwan”.* A contrary opinion reflected by Respondent 259, was that they had “*expected them to be less tame and a bit more wild”.*

Twenty-six respondents (9%) felt that the “*experience exceeded just interacting with the cub, it allowed for reflection*”, whilst only 11 (4%) “*had no expectations*” about the interaction experience. Coded responses of the interactors were significant for the facility (*χ* ^2^_8_ = 23.1; *P* = 0.003), with all groups indicating that the “*interaction was the expectation*” as the leading response.

Respondent 223, a North American, who visited Facility C, felt that the experience had exceeded just interacting and stated that they “*had the opportunity to engage rather than just observe the cubs; they behaved in a natural manner to being rubbed deeply; they weren’t stressed or avoidant, and this tells me they are in a good mental space*”. This also applied to Respondent 245, from Africa, also visiting Facility C, who said that they had “*got to feel their coat, see their personalities, just like our cats at home*”.

One hundred and eighty-three respondents (61%) felt that the experience of interacting with the lion cubs had had no impact on them at all and saw it only as an experience, while 117 respondents (39%) felt that the interaction had been impactful on them. Of those who felt the experience to be impactful, six sets of reasons were provided: Forty-two respondents (36%) described the impact as an “*emotional expression of the experience*”, and 39 (33%) expressed the impact as creating a “*sense of emotion towards the cubs*”. Twenty-five (21%) felt they had “*a desire to support conservation more*” as a result of the impact the interaction had on them. Six (5%) indicated that the impact had resulted in a “*desire to have a cub for themselves*”. Four (3%) respondents indicated a “*religious associated impact*”, with only 1 (1%) saying it had a “*negative impact*”. These coded responses indicated a tendency for the impact felt being affected by the facilities. Those visiting Facility A felt that it elicited a greater “*sense of emotion towards the cubs*”, those visiting Facility B “*a desire to support conservation more*”, and those visiting Facility C more of an “*emotional expression of the experience*”.

Respondent 3, articulated this personal emotional expression as being “*overwhelmed*” while Respondent 9, used the word “*brave*”. Respondent 276 felt “*privileged*” by the experience. There were respondents who reflected on emotions felt towards the cubs, such as Respondent 84, who said “*I feel sorry for them*” and Respondent 109, mentioned “*I feel a twinge of sadness, but maybe some are sacrificed for the greater good of others*”.

**Figure 1.**
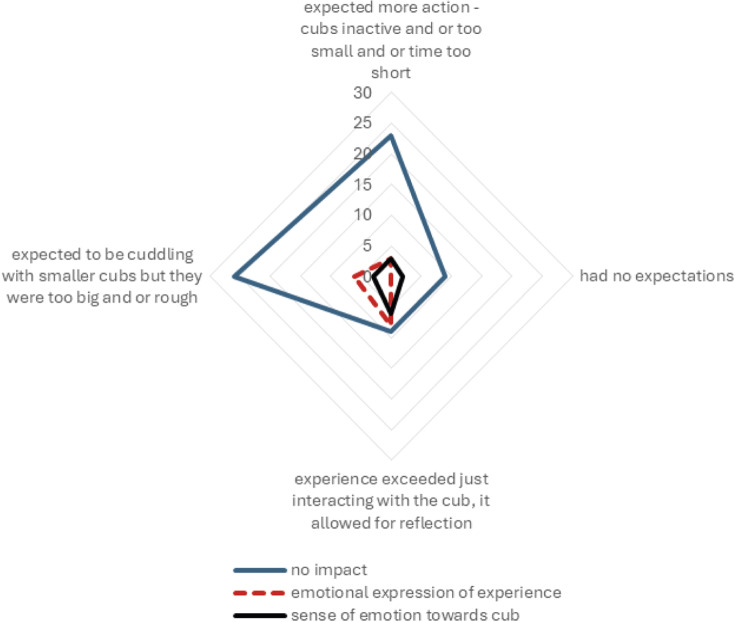
A radar plot depicting the relationship between the prior expectations of the respondents (as shown by the four axes with corresponding n-values) and the resulting impact that the experience had on them (as represented by the legend).

There was a significant relationship between the expectations of the respondents prior to interacting with the cubs and the resulting impact that the experience had on them (*χ* ^2^_24_ = 40.364; *P* = 0.02). When cubs were bigger and rougher than expected or smaller and more inactive than expected, then the experience resulted in having no impact on the interactor (Figure 1).

Of the 300 respondents, only 35 (12%) indicated that they would not interact with lion cubs again, whilst the majority (88%) would if given the opportunity. Of these, 59 (22%) indicated that while they would interact again, it would not be within the next two years. Two hundred and six respondents (78%) indicated that they would participate in the interaction again and were keen to have that repeat experience within two years.

One hundred and sixty (53%) of the 300 respondents also interacted with other animal species whilst visiting the facilities, and these included cheetah (*Acinonyx jubatus*), hyena (*Crocuta crocuta*) cub, rhino (*Ceratotherium simum*), leopard (*Panthera pardus*) and giraffe (*Giraffa camelopardalis*). The favourite animal species for interacting with varied with the facility (*χ* ^2^_15_ = 57.788; *P* < 0.001) due to different animal species being available at the different facilities.

**Figure 2.**
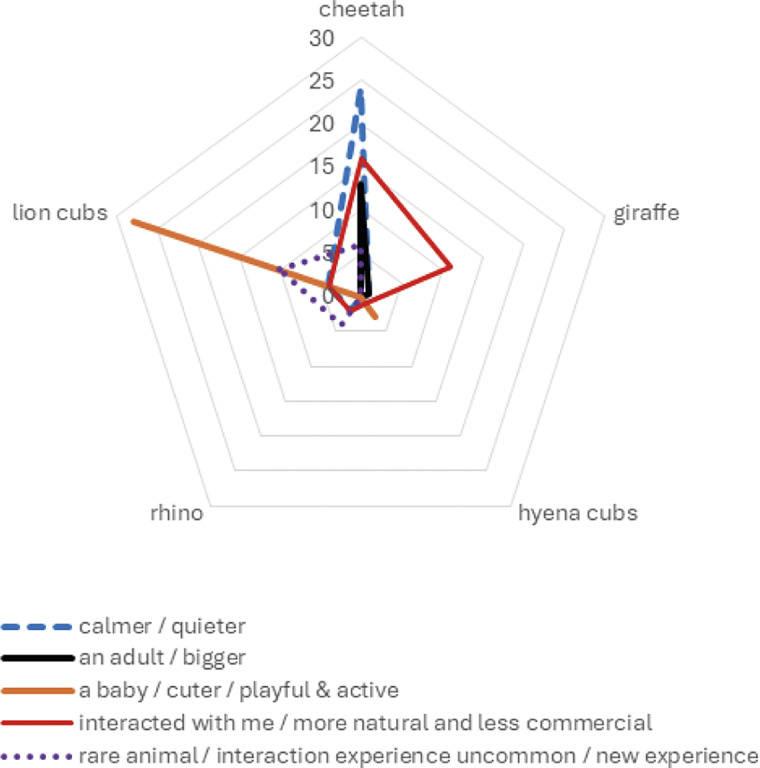
A radar plot depicting the relationship between the animal interacted with (as represented on the five axes with corresponding n-values) and the reason why it was considered a favourite interaction (as represented by the legend).

The reasons provided by respondents for why a specific animal was their favourite to interact with, were significantly associated with the animals’ traits (*χ* ^2^_35_ = 124.574; *P* < 0.0001) (Figure 2). One hundred and forty-six respondents provided reasons as to why a particular interaction with an animal was their favourite and these were coded. The most identified reason, by 34 (23%) respondents, was that the “*animal interacted back with them/felt more natural and less commercial*”, which was most associated with giraffe and cheetah. Thirty-one (21%) reasoned that it was because the animal was a “*baby/cuter/playful and active*”, associated most with lion cubs, with another 31 (21%) saying that it was “*calmer/quieter*”, which was associated most with cheetahs. Twenty (14%) said that it was due to it being a “*rare animal/interaction experience or uncommon/new experience*”. Fourteen (10%) enjoyed it as an “*adult/bigger animal*”, which was associated most with cheetahs, 12 (8%) identified the animal as being “*more dangerous*”. Lastly only four (3%) had felt it was their favourite animal to begin with, thus resulting in it being their favourite interaction experience.

Respondent 191 explained why their favourite interaction had involved a giraffe, saying it was because the animal had “*voluntarily interacted with us and I appreciated that I didn’t force myself on it*; *it* [the interaction] *felt more natural*”. Words used by those who enjoyed the interaction because of the cubs being young, included “*cute*” by Respondent 234 and possessing “*innocence*” by Respondent 115.

Of the 300 respondents interviewed, 147 (49%) were accompanied by children and, of these, 101 (69%) indicated that they had influenced their decision to come and interact. The leading reason (given by 83 respondents [82%]) for how the children had influenced them was best described as “*a desire to experience a wild animal in close proximity and to touch it*”.

Respondent 36 had brought the children to interact “*for the joy they would get and to hold a lion cub, send photos all over the world*” and then questioned “*how many kids get to do that?*” Respondent 207 reasoned “*that it’s* [lion cub interacting] *an experience for them* [children], *otherwise there is just the zoo and you can only see* [animals] *through fences*”. Ten respondents (10%) were “*supporting their child’s love for animals*” through the interaction experience, while nine respondents (9%) viewed the experience as an “*educational opportunity*” for the child, seeing the interaction as a teaching tool.

Respondent 273 explained that their son “*loves animals”*, explaining that “*he doesn’t watch cartoons, but he watches Animal Planet*”. Another parent wanting to support her child’s love of animals was Respondent 35, who stated that “*the five year old is crazy about animals and I want to nurture the passion*”. Respondent 20 said that they “*want them to understand how important nature conservation is and to have compassion for animals*”. Another parent, Respondent 168, felt that it would be a “*unique experience for them, it’s* [the interaction] *not TV, it’s real, nature is a reality and species need to be protected*”.

Responses from the 147 respondents with children accompanying them provided six main sets of responses on how the children had experienced the activity. The majority of children, 57%, “*enjoyed it*”, while 15% were “*nervous/scared/uncertain*”. Ten % found the experience “*impactful*”, while nine percent belonged to a mixed cohort, with “*some children enjoyed it, and some did not*”. Five % were “*disappointed due to limited contact/inactive cubs*” and four % were “*disappointed as denied access*” and this was typically because the child was considered too small to interact.

This negative experience was at times caused inadvertently by the handlers, such as with Respondent 199 who said that the child was “*scared because of what the handler said*” and then quoted the handler as saying “*be careful or they will bite*”. Being ill-prepared for the experience resulted in another negative response. Respondent 96 explained that the child “*didn’t like it* [the interaction] *and wanted to get out* [of the enclosure]”, explaining how one “*didn’t get the same interaction you get from domestic cats*”.

Of the 300 respondents, 173 (58%) felt the interaction to also be an educational experience for them, with a highly significant facility effect (*P* < 0.0001). The coded responses for what was learnt was also highly significant for the facility (*P* < 0.0001), no doubt a result of the different interaction experiences on offer. Eighty respondents (46%) did not learn anything from the guides, but rather “*they learnt indirectly from the experience itself*”. Sixty-four (37%) indicated that “*facts about lions were learnt from the guide*”, 26 (15%) “*learnt how they should conduct themselves around the cubs*”, while only three (2%) mentioned that “*the plight of lions was learnt from the guide*”.

Some respondents had their existing environmental awareness reinforced through the interaction, such as Respondent 19 at Facility A who felt “*a bit more passionate about their conservation*” as result of the interaction. Others, such as Respondent 173, also from Facility A, experienced a change towards his traditional views, saying that he “*grew up killing wild animals, but now things are different, I can save it*, [the] *interaction changed my view*”.

**Figure 3.**
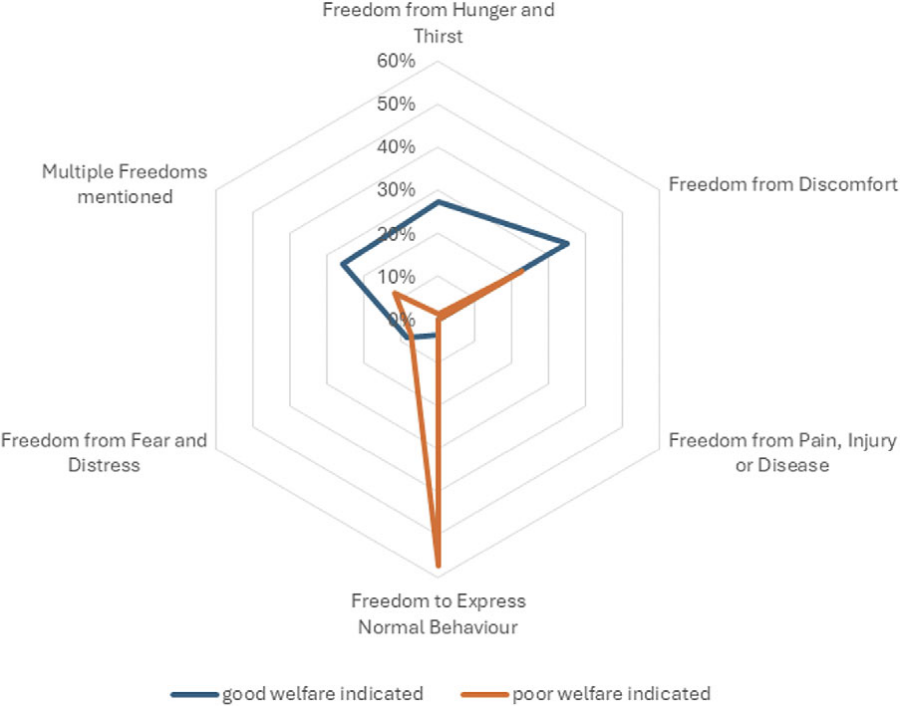
A radar plot depicting the relationship between the Five Freedoms (as represented on the six axes, with corresponding n-values) and its association with a good or poor welfare indicator (as represented by the legend).

Both good and poor welfare identified by respondents was coded according to the Five Freedoms (Farm Animal Welfare Council 1993). Figure 3 indicates the percentages of welfare-identified coded responses. The leading good welfare indicators coded were ‘Freedom from discomfort’ (81 respondents; 35%) and ‘Freedom from hunger and thirst’ (63 respondents; 27%), whilst the leading poor welfare indicators noted a ‘Lack of freedom to express normal behaviour’ (48 respondents; 57%) and a ‘Lack of freedom from discomfort’ (19 respondents; 23%).

Respondent 37, reflecting on positive welfare due to freedom from discomfort, stated that “*The life they* [the cubs] *are leading is fine*, *it is better than what some people have*, *they* [the cubs] *have shelter*”. Respondent 121, reflected differently on poor welfare due to their lack of freedom and discomfort, stating that “*For visitors it is a good experience, but for them* [the cubs], *it is not so good because they have to stay in a little area and wait for visitors. Not good to see them* [the cubs] *pacing, they should be released afterwards, but not sure that this is possible*”. Respondent 36 reflected on poor welfare due to a lack of natural behaviour, stating that the situation was “*Awful and sad. They* [the cubs] *should be in the wild, but their parents are probably also caged. The cubs don’t know what they are missing*”.

## Discussion

This study showed that while 38% of the interactors were aware of controversies surrounding interacting with wildlife and specifically lion cubs, it must be understood that this is not necessarily a reflection of public awareness. It is a limitation of the study that the respondents interviewed did not represent those who are aware of such controversies, choosing to not visit and/or interact. Selfish motives or self-interest often result in people applying their judgement to maximise their own personal objectives (DeScioli *et al*. 2014), as was the case with tourists initiating contact with rehabilitating orangutans (*Pongo pygmaeus*) in Malaysia (Markwell 2001). While this might be a plausible reason in the case of lion cub interactions for those who mentioned the desire to interact despite having knowledge of such controversy, it cannot be assumed that the controversy is always negative and valid. The fact that some respondents indicated their intention to verify the facility for themselves, thus wanting to make up their own opinions about such controversies, suggests that there will always be a market for such activities. If the controversies are to be abated, then a decision will need be made between banning or regulating the industry – a longstanding animal rights debate (Francione & Garner 2010).

It is evident from the responses that the interactors did not have strong expectations when it came to interacting with the lion cubs, as 65% simply expected to interact. This basic need may relate to our dissociation from animals (Curtin 2009), and the act of interacting is seen as a way of getting in touch with nature (Carr & Broom 2018). Marketing and media influence tourist motivations and expectations around animal interactions (Newsome *et al.* 2005). But, understandably, these cubs are very unlikely to be the same individuals as those depicted by the media, and seldom of similar ages, and this may result in expectations not being met. There is therefore a repercussion to facilities using digital and social media to portray the interactions, in that a tourist desires the same experience as that depicted (Carr & Broom 2018). Prior knowledge and attitudes can impact the extent of interactors’ introspection and reflection on their animal experience (Ballantyne *et al.* 2011). In our study, the expectations held by respondents prior to interacting with the cubs were significantly associated with the impact of their experience. This implies that a change in media and advertising could effect a change in expectations, potentially eliciting a more positive pro-conservation impact on the interactors. If interactors had no expectations or were faced with situations that contrast with that which they were hoping for, such as older cubs, when presented with young ones, they reported no impact from the visit. But when expectations were met through the experience, opportunities for self-reflection, empathy for the cubs and a pro-conservation attitude was achieved. Some respondents felt that their expectations were exceeded, as they had had an opportunity to reflect on the actual cubs they had interacted with. This ability to think deeply about an experience or about individuals, i.e. the cubs, allows the respondent to gain a philosophical account of their experience, supports free will and lays the foundations for moral-based values (Velleman 1989). Such responses suggest an understanding of the cubs on a personal and individual level, referring to their states of minds and personalities and not simply objectifying them as a means of entertainment.

The impact of an experience is determined by an affective involvement, and when it is associated with an animal it can result in an environmental social identity, reflecting connectedness, caring and empathy for both animals and nature (Luebke & Matiasek 2013). But, when examining the affective experience responses of respondents, not all reflected a connectedness with the cubs, with several reflecting only on their own affective states. Some respondents expressed empathy towards the individual cubs as opposed to the species, but it is not known whether this would be enough to impart a sense of environmental identity. Conservation awareness was achieved in a few respondents, ranging from a sense of heightened awareness of the plight of wild lions to even changing perceptions around their possible persecution. This awareness is a characteristic of apex predator tourism, but requires support both publicly and politically, through management, monitoring and regulation, if it is to be effective (Macdonald *et al.* 2017). Overall, 21% of the respondents were able to use the impactful experience for positive introspection and reflection, which could lead to them being active conservation advocates (Luebke & Matiasek 2013).

While lion cubs meet the criteria for being favourites amongst visitors, they did not overwhelmingly maintain this position post-interaction, once other animals had also been interacted with. Marginally the most popular interaction experience at Facilities A and C, cubs achieved this on account of their trait for being “*a baby/cuter/playful and active*”, a characteristic known to attract favouritism (Small 2012). The baby schema concept explains how traits associated with young animals have a strong appeal for humans (Borgi *et al*. 2014). This trait appeared therefore to have a greater relationship with the age of the cub rather than the fact that it was a lion. Cheetah were identified as being the favourite animal interaction experience at Facility B for traits which were dissociable from those of the lion cubs, such as being an “*adult*” animal and being “*calmer/quieter*” and for “*interacted back with me/more natural and less commercial*”. Giraffe at Facility C shared this latter trait with the cheetah, as they chose to interact with the public by approaching the fence of their enclosure for feed. Here, the idea that an animal chooses to interact with the public is seen as an attractive trait (Bitgood *et al.* 1988) and was the leading reason why an animal was identified as being the favourite interaction of the day. The psychosocial and psychophysiological effects of human-animal interactions are well described and explain why animal-assisted therapy is so successful (Beetz *et al*. 2012). But it should be noted that a prerequisite for such positive interaction effects, such as those felt through reciprocal interactions, is that the animal is perceived as a social partner thereby allowing for an emotionally relevant relationship (Julius *et al*. 2012).

Half of the respondents were accompanied by children, supporting the idea that lion interaction facilities, as with zoos, are considered destinations for family outings and activities. Wineman *et al.* (1996) classified contact with animals at zoos as high-impact experiences, citing it as an opportunity to overcome fears and cultivate curiosity in children, with similar effects for adults. The majority of these respondents had brought children to interact for an experience associated with proximity and touch. The ability to touch and not just see was mentioned by respondents and clearly set this apart from the experience one could get simply by visiting the zoo. Despite education being a reason provided by 9% of respondents who brought children to interact, it does not imply that only 9% of children would be educated through the experience. Wells and Lekies (2006) suggest that childhood involvement with both wild and domesticated nature can do more than just educate, it can effect change in environmental behaviours. However, Wilson (1995) considers that one of the best ways to teach children about caring for the environment is to practice it, and so respondents who are motivated to educate their children around environmental issues are likely to already be enlightened and pass on this knowledge to their child, not needing the lion cub experience to do so. The rationale of bringing children to interaction activities in order that they might learn or develop a passion for animals is supported by Eagles and Muffitt (1990), who determined that opinion-forming behaviour in children is imperative for the appreciation of wild animals as adults. Whatever the motivation to bring a child to interact, what is more important is how the experience is perceived by the child. A fearful experience could negate the opportunity for positive change in environmental behaviours, as with the children who experienced the interaction negatively due to being nervous, scared and/or uncertain. The children expressed this negative experience as not being suitably prepared for the experience of interacting with the lion cub, incorrectly expecting it to be domesticated or tame. This, at times, also appeared to be perpetuated by comments concerning how the child should behave in order to avoid being injured by the cubs. A state of anxiety causes a child to be unable to process cognitive information efficiently (Perry 1999), thus rendering the experience a lost educational opportunity.

There is a need for both *in situ* and *ex situ* conservation efforts to produce associated outcomes (Buckley *et al*. 2020) and link their existence to environmental problem-solving (Fa *et al*. 2014). Lion numbers have decreased by 43% in the last two decades, with estimates of only 20,000 remaining in the wild (Panthera 2019). Ambassador individuals can facilitate this awareness through close interactions which increase knowledge, have a behavioural effect and create awareness for the animal, its species and nature (Povey & Rios 2002). Understanding this capacity for education, the fact that only just over half of the respondents had felt that they had learnt something is not positive for conservation. Closer inspection of what had been learnt reveals that the majority of the respondents had simply inferred knowledge through their own experiences, such as the feel of the cub’s coat. We can deduce that what the respondent thought they had learnt had only been their perception of an experience. General facts relayed by the guides, including how to conduct themselves around the cubs, does not contribute towards a sense of environmental identity. Only one percent of the total adult respondents had learnt about the plight of lions and were, as a result, equipped to effect a behavioural change.

Fraser (1995) explains how different people consider what is good and/or bad for an animal based on their own judgement about what they consider important for that animal. The lack of differences in attitudes towards welfare explained by the demographics of the respondents is further supported by Herzog and Burghardt (1988), who emphasise that attitudes towards animals are highly personal. Our results suggest there to be overall positive and negative welfare states attributed to lion cubs used in the interaction activities. Freedom from discomfort was the leading positive welfare indicator indicated by respondents. This appeared to be driven by respondents’ feelings, and not a knowledge of welfare, as suggested by Melfi *et al.* (2004). The belief of one respondent that these lion cubs had better lives and shelter than most people, reflects this. Freedom from hunger and thirst was the second positive welfare state indicated by respondents. This is supported by a common notion that, in captivity, animals do not need to seek nor fight for their food and avoid hardships. A lack of freedom to express normal behaviour was the leading reported poor welfare indicator, with respondents identifying the lack of presence of other lions and, in particular, the cubs’ mother. The second leading poor welfare state was the same as the identified leading welfare state, namely discomfort. The reasons given by the respondents were also driven by personal feelings and reflected a belief that the cubs could be leading a better life if they were not used for such interactions. The One Health philosophy considers that there is a need for a healthy and willing animal, mentally stable, to be used in human animal interactions, if there is to be an emotionally relevant relationship from which a positive outcome can be gleaned (Julius *et al.* 2012). This concept seeks interdisciplinary collaboration between human, animal and environmental health care, understanding their interconnectedness (Gibbs 2014). Without optimum welfare, such ambassador lion cubs are unlikely to make the necessary impact on interactors, required for the formation of an environmental identity and to support the survival of their wild conspecifics.

### Animal welfare implications

Animals used in tourist interactions are often termed ambassador animals if they are used in an attempt to educate the public regarding the plight of their species and conservation. The poor welfare of animals used in these interactions however is a well-known and legitimate concern, and it is questionable if they do in fact serve successfully as ambassadors or have the capacity to do so.

Our study found that when interactor expectations are not met by the lion cub interactions, which is often the case if the interaction did not go as anticipated, then the interaction has no effect on the interactor, and the ambassadorial role of the interaction animal is not fulfilled. The study also determined that interactors indicated that they enjoyed interacting with other animals, apart from lion cubs, especially those who voluntarily chose to interact with them, in which case they appreciated the experience more as a result. Our study suggests that the inability to express normal behaviour is the leading poor welfare issue around lion cub interactions. From this, we determine that considerable care is required when selecting animals and environments for interactions, as they are more likely to be successful ambassadors for their wild counterparts and conservation if they are afforded good welfare.

## Supporting information

Wilson and Phillips supplementary material Wilson and Phillips supplementary material

## Supplementary material

The supplementary material for this article can be found at http://doi.org/10.1017/awf.2024.63.
