# Supplementary material for: Tourist perceptions, motivations and expectations when interacting with African lion (Panthera leo) cubs
Source: Anim Welf. 2024 Dec 16;33:e61. doi: 10.1017/awf.2024.63 (PMC11655273; doi:10.1017/awf.2024.63)
Supplement: Wilson and Phillips supplementary material [file S0962728624000630sup001.pdf]

Tourist perceptions, motivations and expectations when interacting with African lion (*Panthera leo*) cubs

Ann Wilson https://orcid.org/0000-0002-2803-446X[a] and Clive JC Phillipshttps://orcid.org/0000-0002-1926-6357[b,c]

[a] Applied Behavioural Ecology and Ecosystem Research Unit (ABEERU), Department of Agriculture and Environmental Sciences, University of South Africa, Private Bag X6, Florida 1710, South Africa,

[b] Institute of Veterinary Medicine and Animal Sciences, Estonian University of Life Sciences, Kreutzwaldi 1, 51006 Tartu, Estonia

[c] Curtin University Sustainable Policy (CUSP) Institute, Curtin University, Australia

Author for correspondence: Ann Wilson, email: cheata@unisa.ac.za

Appendix 1. The questionnaire used in the study to determine the perceptions, motivations and expectations of tourists interacting with African lion cubs.

Part 1:

1. Please indicate your age group:

   ☐ 18 to 30 years ☐ 31 to 50 years ☐ 51 and over

2. Please indicate your continental association:

   ☐ Africa ☐ Australasia ☐ Europe ☐ North America ☐ South America ☐ Asia

3. Please indicate your gender:

   ☐ Male ☐ Female

4. Where do you live:

   ☐ In a city (built up environment with no garden) ☐ Suburbia (housing community with gardens and/or green spaces) ☐ In a rural environment (such as on a farm or game reserve)

Part 2:

5. Are you aware of, or have you heard of any controversy around lion cub interactions?

   ☐ Yes (please answer question 6) ☐ No (please move on to question 7)

6. Despite knowing of the controversy, what made you still come interact today?

Part 3:

7. Would you participate in a cub interaction experience again if you could (assuming you were not affected by distance and/or cost)?

   ☐ No, I have now done it and know what it is about ☐ Yes, but not in the too near future (next 2 years) ☐ Yes, and soon (within the next 2 years)

8. Did you interact with any other animals today?

☐ Yes (please answer question 9) ☐ No (please move on to question 10)

9. Which other animals did you interact with and which animal interaction was the most enjoyable for you and why?

Part 4:

10. Had the actual cub interaction activity not been a part of today's experiences, would you still have visited this facility? And why?
11. Did the actual physical interaction with the lion cub live up to your expectations, please elaborate.
12. What impact if any, did the cub interaction experience have on you?

Part 5:

13. Do you have children under 17 with you here today?

☐ Yes (if yes, then please answer questions 14 & 15) ☐ No (if no, then please move on to question 16)

14. Did the fact that you had children with you here today, in any way affect your decision to come and pet cubs, and why?
15. How was the interaction experience received by the children? Please elaborate:

Part 6:

16. Was the actual interaction an educational experience for you?

☐ Yes (please answer question 17) ☐ No (if no, then please move on to question 18)

17. Please share what you learnt through the actual cub interaction you experienced.

Part 7:

18. What did you think of the welfare of the cubs you interacted with and please elaborate?

Appendix 2. A table showing the strengths of non-significant responses, in associations with 'Facility', 'Age of respondent', 'Continental association', 'Gender' and 'Dwelling', in the questionnaire used in the study to determine the perceptions, motivations and expectations of tourists interacting with African lion cubs.

	Facility	Age of respondent	Continental association	Gender	Dwelling
Interactor was awareness of controversy	$P = 0.72$	$P = 0.13$	$P = 0.25$		
Interactor chose to still interact despite being aware of controversy	$P = 0.10$	$P = 0.98$	$P = 0.46$		
Interactor would still have visited the facility even if lion cub interactions not offered	$P = 0.24$	$P = 0.23$	$P = 0.40$	$p = 0.93$	
Interactor reason for visiting the facility		$P = 0.72$	$P = 0.74$		
Expectations around interacting with the lion cubs		$P = 0.34$		$P = 0.11$	
Impact of the interaction experience	$P = 0.06$	$P = 0.19$	$P = 0.78$	$P = 0.42$	
Favourite animal interacted with		$P = 0.24$	$P = 0.99$	$P = 0.95$	
Children influenced the decision to interact	$P = 1$		$P = 0.77$		$P = 1$
How the child experienced the interaction	$P = 0.74$		$P = 0.94$		$P = 0.77$
Identification of good welfare	$P = 0.46$	$P = 0.12$	$P = 0.23$	$P = 0.99$	
Identification of poor welfare	$P = 0.51$	$P = 0.94$	$P = 1$	$P = 1$	
